# Hernia of the bladder through the broad ligament with renal agenesis and ipsilateral ureter blind ending

**DOI:** 10.11604/pamj.2014.18.218.4906

**Published:** 2014-07-16

**Authors:** Aziz El Madi, Khalid Khattala, Mohammed Rami, Youssef Bouabdallah

**Affiliations:** 1 Department of Pediatric Surgery, Hassan II University Hospital; Fes, Morocco

**Keywords:** Renal agenesis, bladder hernia, broad ligament, ovarian hypoplasia, child

## Abstract

Hernia through a defect of the broad ligament is extremely rare in children. These defects can result from a developmental defect or a spontaneous rupture of cystic formations remnants of mesonephric ducts or Müller. Genital anomalies associated with unilateral renal agenesis are more common in females. We report the case of a 13 months girl allowed for assessment of recurrent urinary tract infection; abdominal examination did not objectified palpable mass, the external genitalia were without abnormalities. Abdominal ultrasound revealed a left renal space is empty with a retrovesical cyst. Cystography was requested that objectified a large pelvic cystic mass retrovesical communicating with the bladder, there was also a left vesicoureteral reflux. Uro- MRI showed a cystic formation retrovesical communicating with the bladder, the right pelvic kidney; uterus is normal size for age. DMSA scintigraphy confirmed the absence of the left kidney with the right kidney that ensures 100% of total renal function. To surgical exploration we found a hernia of the bladder through the left broad ligament, the uterus was dislocated on the right side; left ovary was hypoplasic; the ipsilateral ureter was blind with renal agenesis, we performed by reduction of the bladder then closing the hernial orifice, dissection of the ureter with its ligation and section at the vesical stoma. The postoperative course was uneventful. Evolution is favorable. This observation illustrates a hernia of the bladder through the broad ligament associated with ovarian hypoplasia, renal agenesis and ipsilateral ureter blind ending; this association was not described to our knowledge in the literature.

## Introduction

Genital anomalies associated with unilateral renal agenesis are caused by to agenesis or hypoplasia of the urogenital ridge. They may also be the result of aberrations of the mesonephros in its distal part [1]. The defect of the broad ligament is often acquired, it's obstetric. Congenital origin is due to a developmental defect [2] or spontaneous rupture of cystic formations in this ligament [3], it's extremely rare.

## Patient and observation

A young girl aged 13 months, with a history of recurrent urinary tract infections, admitted to the pediatric department for management of urinary tract infection, Urine culture was isolated Escherichia coli sensitive to cephalosporins and aminoglycosides. Clinical examination found a tonic child, hemodynamically, stable febrile at 38°C, we did not find abdominal mass or abnormalities of the external genitalia.

The biological assessment showed leukocytosis at 18,000 cells/mm3, C-reactive protein at 56 mg/l; renal function was normal with serum creatinine at 6 mg/l and blood urea at 0.3g/l.

**Figure 1:**
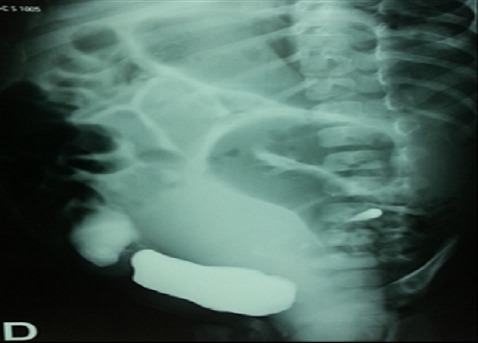
Cystography objective pelvic cystic mass communicating with the bladder

**Figure 2:**
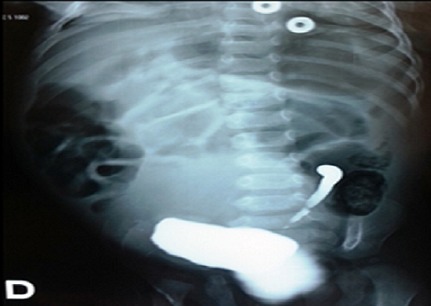
Vesicoureteral reflux on a blind ending ureter

Abdominal ultrasound revealed a hypertrophic right kidney against the left renal space is empty with a retrovesical cystic mass. After treatment of the infection and sterile cytobacteriological examination of urine, cystography was requested that objectified retrovesical opacity communicating with the bladder (Figure 1), there was also a left vesicoureteral reflux (Figure 2). Uro-MRI showed a cystic formation retrovesical communicating with the bladder, pelvic right kidney, and uterus is normal size for age. DMSA has confirmed the absence of the left kidney with the right kidney which ensures 100% of total renal function.

**Figure 3:**
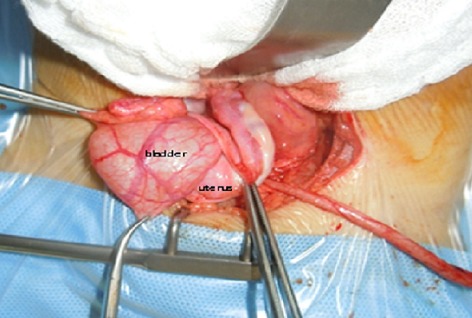
Peroperative picture which shows a herniated bladder back of the uterus. A long blind ending ureter. The right ovary with normal appearance

**Figure 4:**
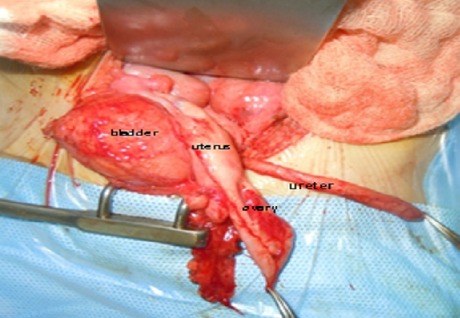
Peroperative picture after reduction of the bladder. Hypoplasic left ovary

Surgical exploration found after a standard Pfannenstiel laparotomy hernia of the bladder through the left broad ligament (Figure 3), the uterus was dislocated on the right with hypoplasic left ovary; ipsilateral ureter blind ending with a left renal agenesis, we proceeded by reduction of the hernia (Figure 4) with a closure defect of the broad ligament. Then dissection of the ureter with ligation and its section at its bladder stoma. The postoperative course was uneventful. The patient was declared coming out of the hospital five days postoperatively; Outcome was favorable.

## Discussion

The unilateral renal agenesis occurs in 0.93 to 1.8 per 1000 autopsies, it is usually diagnosed incidentally during a radiological examination done for another indication [4]. Genital anomalies associated with unilateral renal agenesis occur in 37-60% of women and 12% of men [4]. Hernia of the broad ligament represent 4-5% of all internal hernias and interest only the small bowel in 90%, hernia of the ovary or the ureter is rare [5]. Hernia of bladder through the broad ligament was not described to our knowledge in the literature.

Unilateral renal agenesis is usually asymptomatic when it's isolated. The ureter blind ending has a higher incidence of vesicoureteral reflux than normal ureter and may be manifested by recurrent urinary tract infections as our patient. It can also cause hematuria or forming urinary calculi [6]. O. Okaford and al described a unilateral agenesis of the fallopian tubes and ovaries with ipsilateral renal agenesis in a woman of 36 years [7].

The diagnosis of renal agenesis can be done during the antenatal period, with ultrasound showing an empty lodge without kidney at the abdominal or pelvic level; ultrasonography also find a compensatory hypertrophy of the contralateral kidney. The distal third of the ureter may be dilated with vesico-ureteral junction normal [6].

Alessandrini reported 5 cases of pediatric ureter blind ending; all diagnosed by cystography and he said that this examination should be performed in the first step of investigation of urinary tract in children [8]. Abdominal CT shows when it's a hernia of the intestine which is the most frequent, an agglutination of the small intestine with the air-fluid levels in the pelvic cavity. Intestinal loops repress the rectosigmoid backward and uterus forward.

A classification of the broad ligament hernias has been proposed based on the anatomical position of the defect: Type 1: defect caudal to the round ligament; Type 2: defect above the round ligament; Type 3: defect between the round ligament and the remainder of the broad ligament through the mesoligamentum teres [9, 10].

The treatment consists in reducing the herniated organs and closing the defect. This closure may be performed by laparotomy. Some cases of laparoscopic closure using absorbable clips or separated sutures have been described in the literature [3, 11]. Surgery of the ureter blind ending consists in a classic retroperitoneal resection. Laparoscopic retroperitoneal resection is a minimally invasive alternative reported in adults [12]. The prognosis is favorable if there are no abnormalities on the contrelateral kidney.

## Conclusion

Hernia through the broad ligament is a very rare form of all internal hernias. The radiological diagnosis is difficult in spite of its development; it's often made at a strangulation obstruction because the small bowel is incarcerated; on the other hand associated with renal agenesis a blind ending ureter are asymptomatic unless there is associated a reflux which may be infected as our case. We have not found all these abnormalities associated with hypoplasia ipsilateral ovary described in infants in the literature.
